# Prolonged Self-Resolving Neutropenia Following Asymptomatic COVID-19 Infection

**DOI:** 10.7759/cureus.16451

**Published:** 2021-07-18

**Authors:** Shreya Desai, Javairia Quraishi, Dennis Citrin

**Affiliations:** 1 Internal Medicine, Rosalind Franklin University of Medicine and Science, North Chicago, USA; 2 Hematology and Oncology, Captain James A. Lovell Federal Health Care Center, North Chicago, USA

**Keywords:** coronavirus disease (covid-19), neutropenia, asymptomatic covid-19, leukopenia, bone marrow biopsy

## Abstract

Multiple hematologic complications have been reported as a result of the novel coronavirus disease 2019 (COVID-19) infection. These include leukopenia, lymphopenia, thrombocytopenia as well as increased risk of venous thromboembolism. Neutropenia is a relatively uncommon finding, especially in asymptomatic patients with no other evidence of systemic infection. A young, healthy male undergoing training for the Navy was admitted with rhabdomyolysis following intense physical activity. He was incidentally noted to have severe neutropenia with the white blood cell (WBC) count of 2.1 × 10^9^/L and an absolute neutrophil count (ANC) of 355 cells/μL one month following prior asymptomatic COVID-19 infection. Further evaluation was negative for other infectious processes, nutritional deficiency, or underlying malignancy. Given young age without comorbidities and lack of febrile illness, watchful waiting was recommended in lieu of bone marrow biopsy which resulted in spontaneous resolution of neutropenia and normalization of WBC. The authors argue that although most hematologic complications of COVID-19 are reported in symptomatic patients, asymptomatic patients also appear to have a risk of developing hematologic complications including bone marrow suppression. Watchful waiting may be an appropriate diagnostic approach in such young, healthy individuals.

## Introduction

Neutropenia is defined as an absolute neutrophil count (ANC) less than 1500 cells/µL [1]. The risk of infection increases in individuals with prolonged neutropenia lasting more than seven days and when the ANC is below 500 cells/μL, also defined as severe neutropenia [1]. Multiple hematologic complications have been reported as a result of the novel coronavirus disease 2019 (COVID-19) infection including leukopenia, lymphopenia, thrombocytopenia as well as increased risk of venous thromboembolism [2]. Neutropenia is a relatively uncommon finding, especially so in asymptomatic patients with no other evidence of systemic infection [2]. Here we report a case of an adolescent male with prior asymptomatic COVID-19 infection who presented with severe neutropenia which ultimately resolved spontaneously. 

## Case presentation

A 21-year-old male Navy recruit presented to the emergency department (ED) with bilateral arm pain. The patient was enrolled in the initial stage of the Navy training which required intense physical activity. He denied any infectious symptoms including fever, chills, cough, shortness of breath, or gastrointestinal symptoms. Medical history was remarkable for asymptomatic COVID-19 infection one month prior to presentation. On initial examination, the patient was noted to be afebrile, blood pressure was 130/65 mmHg, and heart rate was 56 beats/min with oxygen saturation of 97% on ambient air. Physical examination was otherwise unrevealing. Laboratory findings were significant for creatine phosphokinase (CPK) 67,873 u/L, aspartate aminotransferase (AST) 219 u/L, and alanine transaminase (ALT) 219 u/L concerning rhabdomyolysis. The patient was admitted to inpatient service for IV fluids and monitoring of arm pain. Repeat COVID-19 testing at this time was negative. Antibodies for severe acute respiratory syndrome coronavirus 2 (SARS-CoV-2) were elevated as expected due to presumed recovery from the virus. Over the course of the next several days, his arm pain resolved with normalization of CPK levels. 

 During the hospitalization, the patient was incidentally noted to have severe neutropenia with a white blood cell (WBC) count of 2.1 × 109/L and an ANC of 355 cells/μL. Further chart review revealed the neutropenia was present at the time of COVID-19 infection one month ago with the WBC 2.5 × 109/L and ANC 415 cells/μL. During the course of his inpatient stay, the patient remained severely neutropenic while the hemoglobin and platelet counts remained within normal ranges. Laboratory studies did not show any evidence of hemolysis. The ultrasound of the spleen showed mild splenomegaly. Given the patient's history of asymptomatic COVID-19 infection, young age and an absence of ongoing infection, watchful waiting was recommended. Bone marrow biopsy was not performed as spontaneous recovery was anticipated. Due to his asymptomatic status and anticipated resolution, granulocyte-colony-stimulating factor (G-CSF) was also not administered. After the resolution of rhabdomyolysis, the patient was discharged with instructions to return should he develop fever or other infectious symptoms. 

**Figure 1:**
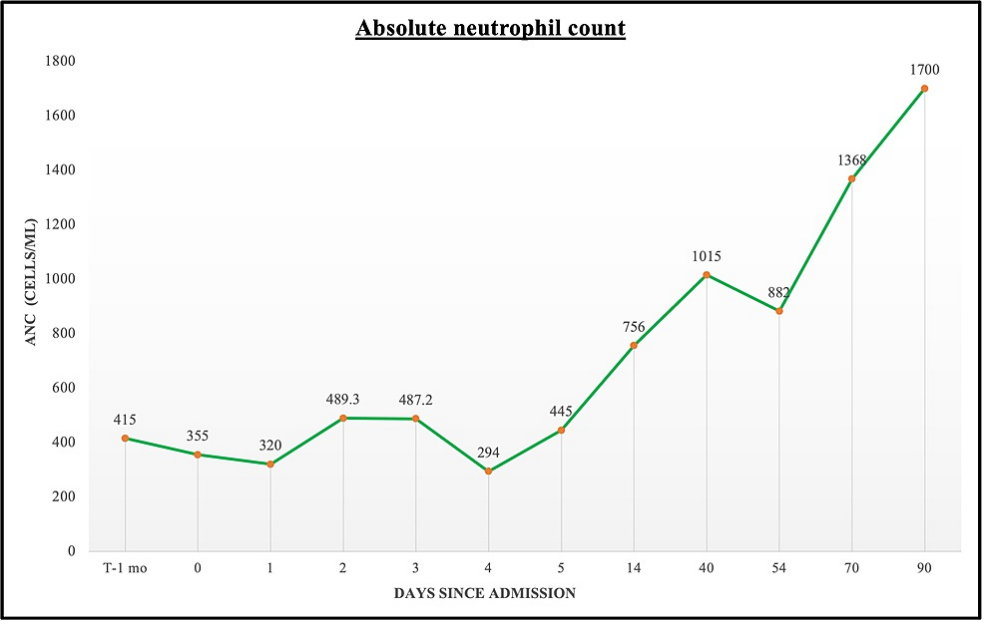
Graphical trend of absolute neutrophil count prior to, during, and following the patient's hospitalization. ANC, absolute neutrophil count

**Figure 2:**
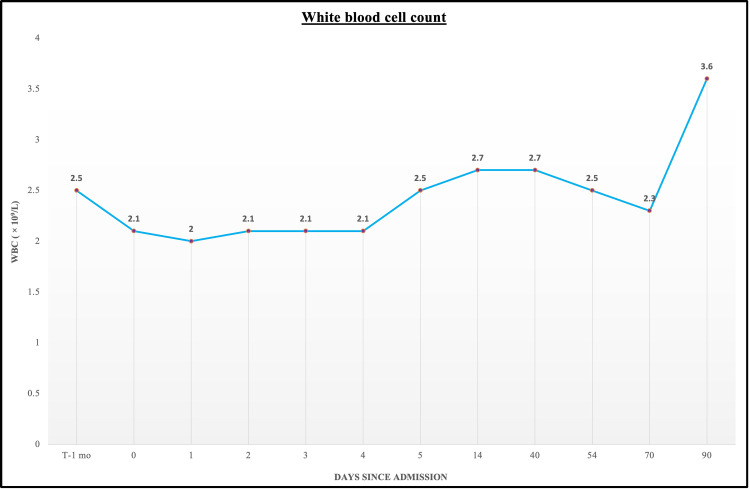
Graphical trend of WBC count prior to, during, and following the patient's hospitalization. WBC, white blood cell

Two weeks after his hospitalization, the patient was noted to have an improvement in the neutropenia with the WBC count of 3.6 × 109/L and ANC 756 cells/μL; antibodies to Epstein-Barr Virus and Cytomegalovirus were negative. Evaluation at three months following his hospitalization showed normalization of the WBC (Figures 1-2).

## Discussion

Neutropenia may be caused by an infection, medication, exposure to a toxin, vitamin B12 deficiency, inflammatory/autoimmune process, and/or malignancy [1]. Our patient's negative blood cultures and viral serologies eliminated the presence of acute or recent infection except for prior COVID-19 infection. Laboratory findings did not reveal evidence of B12 deficiency. Given the patient was otherwise healthy and did not take medications on a regular basis, medications were also excluded as a potential etiology of his neutropenia. The patient's history, young age, physical exam, and normal radioimaging findings diminished the possibility of malignancy.

Various hematologic complications of COVID-19 infection have been reported since the beginning of the pandemic. Hematologic parameters such as lymphopenia, thrombocytopenia, and elevated d-dimer are common in patients with symptomatic COVID-19 pneumonia and carry poor prognosis [2]. COVID-19 can cause a profound inflammatory response and can cause significant immune damage including to the bone marrow. A potential cause of bone marrow suppression may be due to direct infection of the marrow cells leading to abnormal hematopoiesis [3]. Similar bone marrow suppression resulting in neutropenia can be seen with other viral infections such as Influenza viruses, Epstein-Barr Virus, Cytomegalovirus, and parvoviruses [1, 4].

However, the majority of the hematologic abnormalities associated with COVID-19 infection occurred in symptomatic individuals with varying disease activity [2]. Interestingly, our patient did not have any symptoms at the time of COVID-19 diagnosis to suggest a hyper-inflammatory response. However, he suffered from prolonged myelosuppression for four months from the original diagnosis of COVID-19 infection. Due to the absence of infectious symptoms, no antifungal or antibacterial prophylaxis medications were administered. Furthermore, the role of G-CSF in patients with COVID-19 infection remains controversial. Several case reports of cancer patients with COVID-19 infection who received G-CSF were noted to have worsening respiratory status and outcomes [5-6]. In our patient, G-CSF was not considered as he never developed febrile illness while undergoing monitoring of his neutropenia. 

To date, we have been unable to find case reports describing severe, prolonged neutropenia associated with recent asymptomatic COVID-19 infection with spontaneous recovery. However, as most patients with asymptomatic COVID-19 infection would commonly not seek medical care, the incidence of severe neutropenia in such a population is unknown. During the watchful waiting period, our patient's severe neutropenia spontaneously resolved and he remained infection-free. In such young, healthy individuals such as our patients, watchful waiting may be a preferred course of management rather than bone marrow biopsy. 

## Conclusions

COVID-19 infection is associated with various hematologic complications, particularly in severely ill patients. However, as our case report highlights, even asymptomatic patients appear to be at an increased risk of developing bone marrow suppression due to the virus despite the lack of overt inflammatory response. In healthy, asymptomatic individuals without febrile illness, watchful waiting ought to be considered as the primary management prior to performing a bone marrow biopsy. In high-risk individuals such as nursing home residents, more stringent isolation precautions may be considered while monitoring of the neutropenia. 
